# Design and Control of an Embedded Vision Guided Robotic Fish with Multiple Control Surfaces

**DOI:** 10.1155/2014/631296

**Published:** 2014-01-29

**Authors:** Junzhi Yu, Kai Wang, Min Tan, Jianwei Zhang

**Affiliations:** ^1^State Key Laboratory of Management and Control for Complex Systems, Institute of Automation, Chinese Academy of Sciences, Beijing 100190, China; ^2^Department of Informatics, University of Hamburg, D-22527 Hamburg, Germany

## Abstract

This paper focuses on the development and control issues of a self-propelled robotic fish with multiple artificial control surfaces and an embedded vision system. By virtue of the hybrid propulsion capability in the body plus the caudal fin and the complementary maneuverability in accessory fins, a synthesized propulsion scheme including a caudal fin, a pair of pectoral fins, and a pelvic fin is proposed. To achieve flexible yet stable motions in aquatic environments, a central pattern generator- (CPG-) based control method is employed. Meanwhile, a monocular underwater vision serves as sensory feedback that modifies the control parameters. The integration of the CPG-based motion control and the visual processing in an embedded microcontroller allows the robotic fish to navigate online. Aquatic tests demonstrate the efficacy of the proposed mechatronic design and swimming control methods. Particularly, a pelvic fin actuated sideward swimming gait was first implemented. It is also found that the speeds and maneuverability of the robotic fish with coordinated control surfaces were largely superior to that of the swimming robot propelled by a single control surface.

## 1. Introduction

There has been a rapid increase in the research and development of the bioinspired mechatronic system in academia and industry over the last 20 years, particularly due to the increasing vitality in biomimetics [1]. As a small branch, extensive work on designing and controlling various bioinspired swimming robots has been done since the first fish-like robot, RoboTuna, was developed at MIT [2]. By understanding and adapting the underlying principles of swimming motions of aquatic animals (e.g., fish and cetaceans) to artificial swimming machines (hereafter termed robotic fish), an enhanced comprehensive performance has been obtained [3–7]. It is expected that a silently moving, agile robotic fish will be more competent than current autonomous underwater vehicles propelled by rotary propellers. Once well developed, the robotic fish could carry out a range of tasks, such as underwater exploration, oceanic supervision, patrol, pollutant search, and building underwater mobile sensor networks.

As an alternative propulsion device, fins (or hydrofins) are widely used by aquatic animals. The propulsion modes of swimming fish, from a functionality standpoint, are classically divided into body and/or caudal fin propulsion (BCF) and median and/or paired fin (MPF) propulsion [8]. Specifically, fish classes that use varying degrees of body undulation and/or caudal fin oscillations for thrust generation are examples of the BCF mode, while fish relying on paired fins (pectoral fins and pelvic fins) and/or median fins (dorsal fin and anal fin) for thrust generation are categorized under the MPF mode. It is generally thought that no absolutely superior model exists among these modes since body shape and motor function level closely depend on the fish habitats.

More recent evidence has suggested that fish actually rely on multiple control surfaces including the caudal fin, the pectoral fins, the pelvic fins, the dorsal fin, and the anal fin as well as the body to achieve fast and maneuverable propulsion [9]. Particularly, the position, mobility, and hydrodynamic characteristics of the control surfaces are linked with the swimming performance. These well-integrated, configurable multiple control surfaces provide an excellent paradigm to create and control high-performance underwater vehicles [10]. However, it is unrealistic to totally replicate a natural fish due to the tremendous difference between the biological system and the engineering counterpart. Tradeoffs in engineering practice will have to be struck among biological mechanism, engineered method, feasibility, cost/gain, and so forth. The existing robotic fish, however, have predominantly used BCF or pectoral fins (PF) or undulatory fins (UF) for propulsion and maneuvering. There have been few or limited studies related to simulating and constructing a robotic fish with multiple different fins, which are desirable for enhanced controllability and maneuverability. For example, inspired by the boxfish with multiple fins, Lachat et al. designed a miniature boxfish-like swimming and crawling robot with three actuated fins [11]; Kodati et al. developed a robotic ostraciiform with a self-correcting mechanism to emulate the maneuverability of the boxfish [12]. In these two boxfish-like robots, only two types of fins including pectoral fin and caudal fin were incorporated and examined, making them unavailable for the extensive exploration of various fin-fin and body-fin interactions.

On the other hand, nearly all daylight fish have color vision that is at least as good as a human's [13]. Although fish do not use vision as one of their primary senses, underwater vision is of particular significance for predator-prey interactions in the high-contrast, high-chroma, and cluttered aquatic environments. Using visual information as a clue, a vision-based robotic fish can respond to the dynamic change of the environment and mediate its movements accordingly. However, locomotion and visual information are generally treated as separated problems in the field of robotics. Recent studies reveal that the locomotion and vision should closely interact. This interaction is crucial for autonomous and adaptive control. For example, Gay et al. applied a combination of vision and central pattern generator (CPG) to perform a reaching task for a nonholonomic crawling humanoid robot [14]. Although vision-based applications for terrestrial robots have been extensively explored, autonomous goal-directed swimming control of robotic fish using only visual information is rarely tackled. Fortunately, with the aid of embedded systems, many kinds of application-specific embedded vision systems have emerged. The performance of embedded vision systems is much improved with the advancement of embedded technology, offering an integrative, small-sized, low-cost vision-based control solution [15].

This paper, on the basis of our previous research on fish-like swimming [16, 17], aims at creating a self-propelled robotic fish with multiple complemented gaits and improved swimming performance. Specifically, a caudal fin, a pair of pectoral fins, and a pelvic fin are appropriately incorporated into a free-swimming platform and are cooperatively operated. With onboard embedded vision functioning as a receptor, the robotic fish can decide its motion autonomously. Note that the proposed robotic fish design is loosely inspired by a class of fish (e.g., bluegill and Ostraciidae) that use multiple fins in many different gaits acting on an unstable body to move through their aquatic environments [18]. In particular, a specific sideward swimming was first implemented with the reciprocally oscillating pelvic fin, in which the robot averaged a sideways speed of 6.5 ± 0.4 cm/s. Compared with the swimming gait involved a single control surface, the gait involved coordinated control surfaces exhibited increased speeds and better turning performance. In addition, the successful implementation of goal-directed search and tracking demonstrated the efficacy of the integration of vision and CPG-based motion control for online underwater navigation.

The remainder of the paper is organized as follows. In Section 2, the robotic fish prototype is introduced by describing its mechatronic configuration and motion control. The embedded vision centered navigation method is elaborated in Section 3. Experiments and results are provided in Section 4. Finally, conclusions and future work are summarized in Section 5.

## 2. Mechatronic Design and Motion Control

In this section, we will restrict our attention to the main problem of mechanical design and swimming kinematics as well as motion generation and modulation for a self-propelled, multifin robotic fish suited for shallow waters.

### 2.1. Mechatronic Design

**Figure 1:**
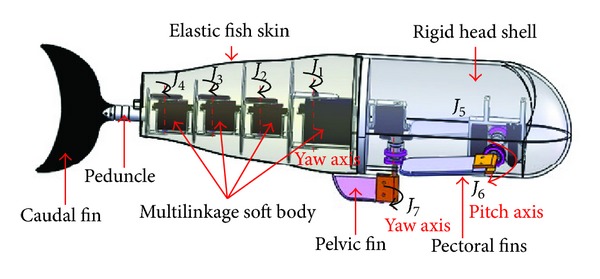
Mechanical drawing of a self-propelled robotic fish with multiple control surfaces.

As stated previously, efficient and agile swimming motions are dominantly attributed to coordinated movements of multiple control surfaces involving the body and the accessory fins. Different fins, in principle, have different functions, which complement one another. More exactly, the body plus caudal fin are responsible for propulsion and steering. The pair of pectoral fins lends itself to locomotion and side-to-side motions. The pelvic fin is extremely helpful in balancing the fish for propulsion, steering, and paddling. Many fishes switch between modes involving various fins, primarily depending on whether fast or slow swimming or hovering is demanded. In particular, most fishes have median fins that can be erected or depressed, adding a dynamic quality to their locomotion [10]. Loosely inspired by a class of fish (e.g., bluegill and Ostraciidae) that use multiple fins in many different gaits acting on an unstable body to move through their aquatic environments, a new multi-fin robotic fish illustrated in Figure 1 is proposed. Unlike the boxfish-like robots implementing ostraciiform or labriform swimming modes [11, 12], the conceived robotic fish is designed to perform carangiform swimming via the body plus caudal fin, labriform swimming via the pectoral fins and UF mode via the pelvic fin.

Mechanically, the designed robotic fish consists of a rigid head, a multilinkage soft body, a peduncle and caudal fin, a pair of pectoral fins and a pelvic fin. Notice that the pelvic fin is used instead of the anal fin for sideward maneuverability and for ease of installation and waterproof protection. More specifically, the multi-linkage soft body consists of four servomotors connected in series with aluminium links, providing four independent joints (*J*_1_–*J*_4_) around the yaw axis. The outside of the multi-linkage soft body is wrapped by a compliant, crinkled rubber tube functioning as fish skin. It is worthwhile to note that the output torque of servomotors should increase from tail to head, so the driving motor for the first link has a bigger torque. Considering the caudal fin, in its final lash, may contribute as much as 40 percent of the forward thrust in carangiform and thunniform swimming [19]; a crescent-shaped caudal fin is connected to the last link via a slim peduncle made of polyvinyl chloride. The caudal fin is made of partly compliant material, polyurethane, for producing more thrust [20].

**Figure 2:**
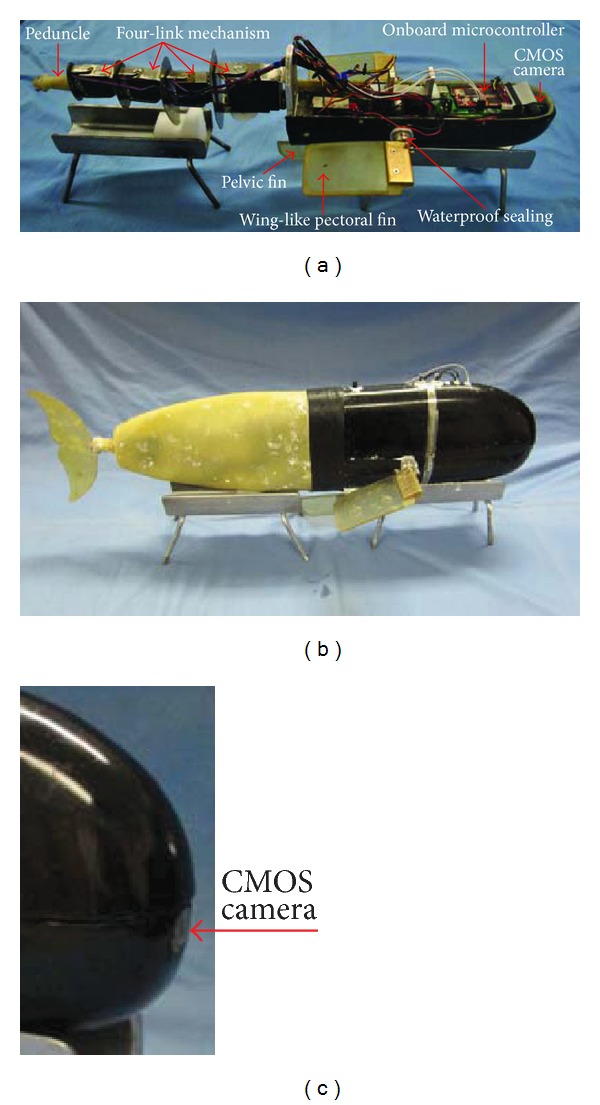
Photograph of the developed robotic prototype. (a) The assembled prototype. (b) The final prototype. (c) A feature of the onboard camera.

Concerning the accessory fins, as shown in Figure 2(a), the pectoral fins are two oscillating foils laterally mounted at the rear lower position of the rigid shell, providing two independent joints (*J*_5_ and *J*_6_) around the pitch axis. Note that each pectoral fin, capable of 0–360° rotation via a set of custom-built gears, can be controlled synchronously or independently. The pelvic fin located at the posterior bottom of the fish shell provides a single DOF joint (*J*_7_) around the yaw axis. The imported pelvic fin plays a role like a rudder on a ship. For the purpose of waterproof protection, the rotations of the servomotors in these accessory fins are transmitted to the outside through a dynamic waterproof sealing. Currently, the oscillatory angles of *J*_1_–*J*_4_ are restricted to ±60° that of *J*_7_ is limited to ±90°, whereas those of *J*_5_ and *J*_6_ are expanded to ±180° allowing for both forward and backward swimming. Such an adequately large oscillation range permits large-amplitude and fast swimming modes.

Figure 2(b) shows the developed multifin robotic prototype. The prototype has a dimension of 0.68 m × 0.26 m × 0.22 m (*L* × *W* × *H*) and weighs approximately 5.20 kg. Both pectoral fins have the same dimensions, that is, 12 cm in length, 8 cm in width, and 0.5 cm in height. Notice that, to achieve a balance between buoyancy and weight, the distribution of mechatronic components has to be carefully arranged. Due to the lack of an automatic adjusting apparatus equivalent to the swim bladder in real fish, a neutral buoyancy state is almost achieved by careful calculations and balancing.

In addition, to facilitate the implementation of embedded vision-based underwater navigation, a pinhole CMOS camera is mounted with a transparent window glued to the most anterior of the rigid head (see Figure 2(c)). The image data is acquired from the image sensor (i.e., OV7620) capable of RGB and YUV format output. The camera module C3188A mainly comprises an image sensor with a 4.9 mm lens, few passive components, and a crystal at 17.7 MHz. The used control module is a microcontroller (Samsung S3C2440) encompassing a high-performance 32-bit RISC, ARM920T CPU core running at 400 MHz, and a series of peripherals. The onboard memory involves 64 MB SDRAM used during programming and 64 MB Nand Flash for permanent data and code storage. Functionally, the embedded control module is responsible for generating fish-like swimming control signals coded in pulse width modulation (PWM) waveform of oscillatory servomotors and for real-time image processing designed to perceive the aquatic environments.

### 2.2. CPG-Based Swimming Control

To mimic the BCF-type swimming, a traveling fish body wave that appears as a curvature of the spine and muscle with increasing amplitude is usually exploited. A widely used body wave is described by

(1) $$\begin{array}{c} y_{\text{body}}\left(x,t\right)=\left(c_{1}x+c_{2}x^{2}\right)\sin\left(kx+\omega t\right), \end{array}$$

where *y*_body_ represents the transverse displacement of the moving tail, *x* denotes the displacement along the main axis, *k* indicates the body wave number (*k* = 2*π*/*λ*), *λ* is the body wave length, *c*_1_ is the linear wave amplitude envelope, *c*_2_ is the quadratic wave amplitude envelope, and *ω* is the body wave frequency (*ω* = 2*πf* = 2*π*/*T*). In [4], a discretized body wave design method is proposed. That is, the oscillatory body is discretely designed as a multilinkage (or *N*-link) mechanism composed of several oscillating hinge joints actuated by motors. The discrete form of (1) is given by

(2) $$\begin{array}{c} y_{\text{body}}\left(x,\kappa \right)=\left(c_{1}x+c_{2}x^{2}\right)\sin\left(kx\pm \frac{2\pi }{M}\kappa \right), \end{array}$$

where *κ* denotes the *κ*th variable of the sequences *y*_body_(*x*, *κ*) (*κ* = 0,1,…, *M* − 1) in one oscillation period and *M* indicates the discrete degree of the overall traveling wave.

For the sake of simplicity, the movements of control surfaces are considered as oscillatory in a harmonic (sinusoid) fashion that can be described as follows:

(3) $$\begin{array}{c} \theta_{i}\left(t\right)={\bar{\theta }}_{i}+A_{i}\sin\left(2\pi f_{i}t+\phi_{i}\right)\;\left(i=1,2,\ldots ,7\right), \end{array}$$

where *θ*_*i*_(*t*) represents the angular position of the *i*th control surface at time *t*, ${\bar{\theta }}_{i}$ indicates the angular bias for asymmetric oscillations deviating from the median position, *A*_*i*_ stands for the oscillatory amplitude, *f*_*i*_ is the oscillatory frequency, and *ϕ*_*i*_ is the phase difference. As a response to the input data, the swimming speed can be mediated by the magnitude of amplitude (*A*_*i*_) and frequency (*f*_*i*_), while the direction is influenced by the bias (${\bar{\theta }}_{i}$). The control commands are then wirelessly transmitted to the robotic fish in a remote control manner.

**Figure 3:**
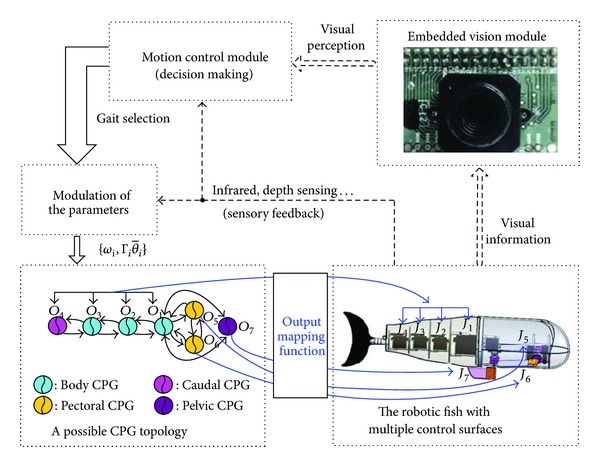
Diagram of the complete control architecture.

However, this body wave-based swimming control should be properly discretized and parameterized for a specific swimming gait [4]. Moreover, the swimming stability of a desired gait and the smooth transition between two different gaits are hardly guaranteed, which is unfavorable to steady image data acquisition. Hence, other approaches should be sought to grab a steadily moving image and thus to alleviate the burden of embedded computation while not affecting the accuracy. Inspired by the lamprey, an eel-like fish whose propulsion is governed by activity in its spinal neural network; some CPG-based models have been built to produce fish-like swimming. As summarized by Ijspeert, CPG-based control presents several attractive features including distributed control, the ability to deal with redundancies, fast control loops, and permitting the modulation of locomotion by simple control signals [21]. For the robotic fish with multiple control surfaces, as shown in Figure 3, a complete CPG-centered control architecture is established. By coupling a set of nonlinear oscillators, a CPG network is built, involving one caudal CPG (*O*_1_), three body CPGs (corresponding to *O*_2_–*O*_4_), two pectoral CPGs (*O*_5_ and *O*_6_), and one pelvic CPG (*O*_7_). Each swimming gait is then encoded with a standardized “template” corresponding to a CPG parameter set. With the proper tuning of feature parameters, diverse swimming gaits in three dimensions results. Notice that, in the interests of simplicity, a weak coupling scheme is adopted, where all self-couplings are eliminated. Considering that the pectoral fins and the pelvic fin can be individually controlled, the couplings to *O*_5_, *O*_6_, and *O*_7_ are not intrinsic but optional. The dynamics of the *i*th oscillator is described as follows [22]:

(4) $$\begin{array}{c} {\dot{u}}_{i}=-\omega_{i}v_{i}+u_{i}\left(\Gamma_{i}^{2}-u_{i}^{2}-v_{i}^{2}\right)+\overset{n}{\underset{j=1,j\neq i}{\sum }}a_{ij}v_{j},\\ {\dot{v}}_{i}=\omega_{i}u_{i}+v_{i}\left(\Gamma_{i}^{2}-u_{i}^{2}-v_{i}^{2}\right)+\overset{n}{\underset{k=1,k\neq i}{\sum }}b_{ik}u_{k}, \end{array}$$

where *n* indicates the total number of nonlinear oscillators in the CPG network, having *n* = 7 in the paper. The state variables *u*_*i*_ and *v*_*i*_ denote the membrane and adjustment potential, respectively. *ω*_*i*_ and Γ_*i*_ stand for the intrinsic oscillatory frequency and amplitude. ∑_*j*=1,*j*≠*i*_^*n*^ *a*_*ij*_ *v*_*j*_ and ∑_*k*=1,*k*≠*i*_^*n*^ *b*_*ik*_ *u*_*k*_ are the coupling relationships of the *i*th oscillator with other oscillators in the CPG network. *a*_*ij*_ and *b*_*ik*_ are the corresponding coupling weights. It should be emphasized that this coupling is just an assumption of the CPG model and that whether the coupling scheme relates to biological existence or optimality is currently unproved.

To translate the rhythmic output signal of the *i*th oscillator to the motor actuating signal, a mapping function *f*_*i*_(*u*_*i*_) is defined as follows:

(5) θmi=fi(ui)={γiumax⁡i+ubi,ui≥umax⁡i,γiui+ubi,0<ui<umax⁡i,0,ui=0,

where *θ*_*m*_^*i*^ is the driving signal fed to the motor, *γ*_*i*_ is the transformation coefficient, *u*_max⁡_^*i*^ is the upper bound for the driving signal of the *i*th oscillator, and *u*_*b*_^*i*^ is the potential bias when the *i*th control surface stays at its reference position. For our robot, the reference positions of the body and the caudal fin and the pelvic fin are in the sagittal plane, and those of the pectoral fins are in the horizontal position.

**Figure 4:**
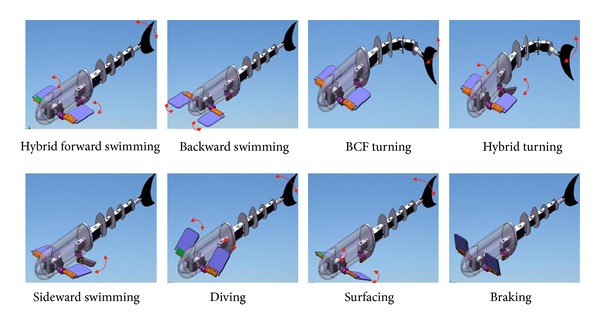
Schematic illustration of typical swimming gaits.

Since the fish-like swimming is regarded as sinusoidal motions, the interactions of different control surfaces can be attributed to coupled factors among CPG units. According to our proposed CPG parameter determination method in accordance with the traveling body wave [23], a set of feature parameters for the fish CPG network can be sought. Thus, through the coordinated control of body CPG, caudal CPG, pectoral CPG, and pelvic CPG, a diversity of swimming gaits such as coordinated forward swimming, backward swimming, hybrid turning, sideward swimming, diving/surfacing, and braking are implemented (see Figure 4).  Table 1 summarizes the relationships between the swimming gaits and the involved control surfaces. For all these gaits, the swimming speed can be altered by adjusting the frequency *ω*_*i*_ and/or the amplitude Γ_*i*_. Meanwhile, the angular bias ${\bar{\theta }}_{i}$ is used for turning maneuvers and 3D swimming. Typically, $15^{{}^{\circ}}\leq {\bar{\theta }}_{1}={\bar{\theta }}_{2}\leq 45^{{}^{\circ}}$ is set for the BCF turning; ${\bar{\theta }}_{5}={\bar{\theta }}_{6}=180^{{}^{\circ}}$ for the MPF backward; $0<{\bar{\theta }}_{5}={\bar{\theta }}_{6}<90^{{}^{\circ}}$ for the PF-based diving; and $-90^{{}^{\circ}}<{\bar{\theta }}_{5}={\bar{\theta }}_{6}<0$ for the PF-based surfacing. It is critical to find appropriate CPG patterns that are closely associated with viable swimming gaits. In this study, a dynamic model of robotic fish swimming using Kane's method is firstly developed to guide the primary parameter search. A particle swarm optimization algorithm is further utilized to yield a relatively optimal parameter set [24]. The main control parameters that are empirically determined with the help of dynamic simulations are listed in Table 2. Note that an assumption is made in simplifying the CPG couplings that the descending weights are identical, implying that the *i*th CPG receives the influence from the (*i* − 1)th one. Namely, *a*_*i*,*i*−1_ = *a*_1_ and *b*_*i*,*i*−1_ = *b*_1_, where *i* = 2,3,…, *n*, so do the ascending coupling weights, holding *a*_*i*,*i*+1_ = *a*_2_ and *b*_*i*,*i*+1_ = *b*_2_. Note also that although vision, infrared, and depth sensing are involved in the control loop (see Figure 3), only embedded vision serves as sensory feedback for online underwater navigation in this paper.

## 3. Embedded Vision-Based Navigation

### 3.1. Embedded System Architecture

**Figure 5:**
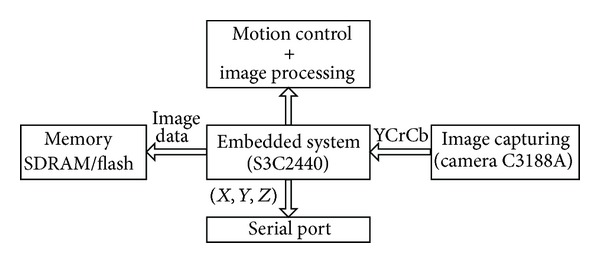
Hardware architecture of the adopted embedded system.

The adopted embedded system is based on the core of ARM9. Basically, the hardware architecture consists of three functional modules, that is, the image capturing module, the image processing module, and the control module. A general schematic view of the system architecture is depicted in Figure 5. The CMOS camera captures the image information at a resolution of 320 × 240 pixels, transforms the optical signal into a digital image signal, and then sends the signal to the memory device SDRAM. The ARM processor gets the image data from the memory device and then processes it according to the algorithms needed. The camera connects with the ARM processor board by the camera interface which is a 32 pins port and the camera functions are programmable through an I2C interface. The ARM processor has an external memory controller, so the SDRAM control can be realized by it. The ARM processor board can be used as not only the host controller for generating various swimming gaits, but also a smart visual perceptor. For the former, the board includes some PWM interfaces to driving motors and other peripherals. As for the latter, the board can return image processing results for subsequent control applications via a serial interface.

At the software level, a “bare metal” ARM system runs on the robotic fish. As usual, the software inside the ARM board consists of a boot program and custom-built application codes mainly involving motion control and image processing. As for the PC part console, a visual and easy-to-use GUI console based on Microsoft Visual C++ has been developed. Via a duplex wireless connection, the user can not only flexibly modulate the swimming motions by altering the CPG configuration, but also conveniently display the visual scene from the onboard CMOS camera.

### 3.2. Vision-Based Navigation

It is generally considered that underwater imagery is partly plagued by many factors involving refraction, absorption, forward scattering, backward scattering, and poor visibility, making the relatively matured computer vision approaches useless sometimes [25]. In this paper, an in-lab aquatic environment is considered in the interest of simplicity. That is, the underwater experiments are performed in an indoor swim tank with clear water. Like the vision-based perception framework for fish motion proposed in [26], the involved embedded vision can primarily be divided into three parts: object segmentation, object detection, and visual tracking. For simplicity, the object to be identified is a rectangular color patch during experiments. The object segmentation and detection can then be accomplished by checking the interested color patch rapidly and accurately.

Regarding object segmentation, a color image segmentation algorithm based on the YCrCb threshold is adopted. Since the color space of YCrCb is widely used for color object segmentation, the image in the YCrCb 4 : 2 : 2 format is applied. Specifically, color image segmentation to the histogram threshold is applied to the color component Cr and Cb. Only if Cr and Cb components are located at the range relatively at the same time as given in (6) does an image point belong to the target color patch.

Consider

(6) Inner  pixel Cr_Lower≤Cr≤Cr_Upper  Cb_Lower ≤Cb≤Cb_UpperBeside  pixel else,

where Cr represents the image point of the red component; Cb denotes the image point of the blue component; Cr_Lower and Cr_Upper stand for the lower and upper boundary of the Cr component; Cr_Lower and Cr_Upper indicate the lower and upper boundary of the Cb component. Note that these two color thresholds are empirically determined by offline experiments.

In the following object dictation, the color centroid of the relevant color patch is computed. That is, a data set of {*X*, *Y*, *Z*} can be obtained, where *X* and *Y* represent the coordinates of a colored object center, respectively and *Z* indicates the pixel number in the interested color patch.

As for the visual tracking, the mean shift algorithm is adopted owing to its computational efficiency and robustness to nonrigid deformation [27]. In essence, the mean shift algorithm is a nonparametric technique that climbs the gradient of a probability distribution to find the nearest dominant mode. Currently, a modified version of the mean shift algorithm, the continuously adaptive mean shift (Camshift) algorithm, is being widely used for head and face tracking. The prominent advantage of the Camshift algorithm is that it can tackle dynamically changing color probability distributions, making it well suited to underwater target tracking. More specifically, a search window that surrounds the target is used to specify the location and size (or scale) of the target in the implementation of the Camshift algorithm. In this paper, the mean location and size of the search window serves as the output of the visual tracking algorithm.

**Figure 6:**
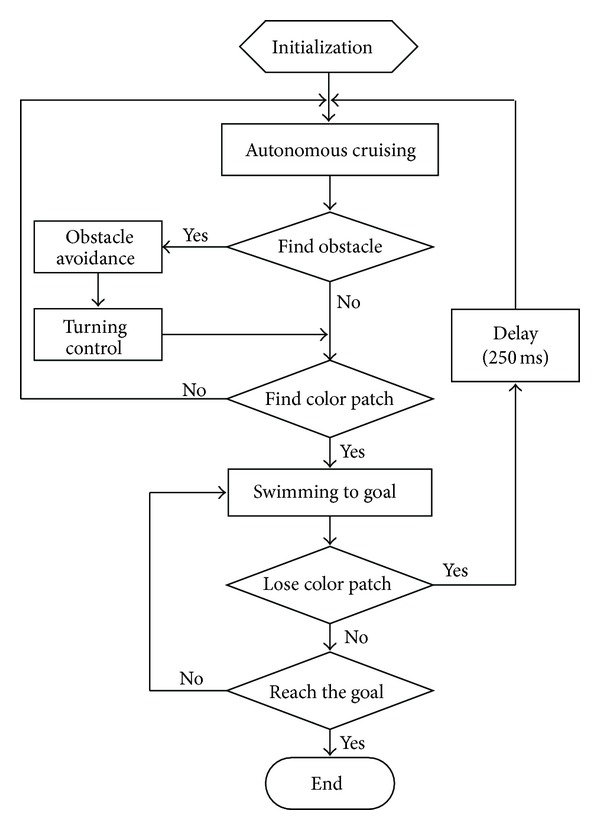
Flowchart of embedded vision-based navigation strategy.

With the embedded vision as the perceptual device of the surroundings, a navigation strategy is exemplified in Figure 6. In particular, two swimming behaviors will be elaborated.

**Figure 7:**
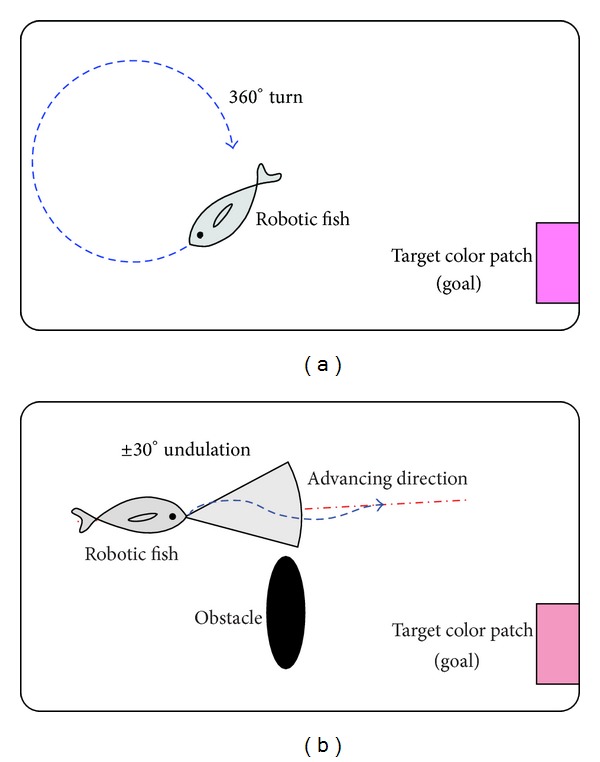
Two autonomous cruising strategies. (a) 360° turn. (b) ±30° undulation.

**Figure 8:**
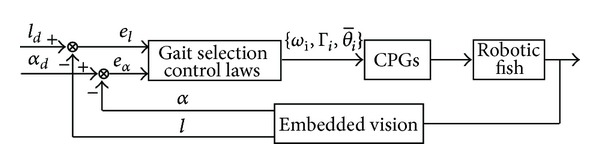
Block diagram of visual feedback coupled closed-loop control in 2D swimming.

- *Autonomous Cruising*. In this behavior, the robotic fish continuously scans the surroundings to detect the target color patch. To avoid a possible blind area of the visual field, as illustrated in Figure 7, a 360° turn-based cruising and a ±30° undulation-based cruising can be combined. The former is intended to obtain a 360° panorama, while the latter is to swim swiftly and straight so that the robotic fish finds the goal as soon as possible.
- *Swimming to Goal*. This behavior activates once the goal is detected. However, because of the effect of inertial drift and induced waves, the robotic fish hardly stops immediately and precisely. So some complementary gaits can be combined to achieve the goal by exploiting information of the distance (*l*) between the robot and the goal as well as the offset angle (*α*) between the horizontal line parallel to the robot and the line connecting the goal and the robot visual field center. As illustrated in Figure 8, the robotic fish can adapt its swimming gait according to visual information that modifies the CPG feature parameters $\left\{\omega_{i},\Gamma_{i},{\bar{\theta }}_{i}\right\}$. Thus a closed-loop control is attained. Here, the distance error (*e*_*l*_ = *l*_*d*_ − *l*, where *l*_*d*_ represents the desired swimming distance) is utilize to vary the swimming speed via *ω*_*i*_ and/or Γ_*i*_, while the heading error (*e*_*α*_ = *α*_*d*_ − *α*, where *α*_*d*_ represents the offset angle when the goal lies in the center of visual field) is employed to trigger turns via ${\overset{-}{\theta }}_{i}$. According to different levels of *e*_*l*_ and *e*_*α*_, different control laws (or fuzzy rules) can be designed [4]. For instance, (1) when *e*_*l*_ is large (e.g., larger than one body length, 1 BL), a fast swimming gait (e.g., BCF forward or hybrid forward) will be used; (2) when *e*_*l*_ is small (e.g., smaller than 0.5 BL), a low yet steady swimming gait (e.g., MPF forward) will be utilized; (3) when *e*_*α*_ is large (e.g., larger than 90°), a large-angle turning maneuver (e.g., BCF turning) will be performed; (4) when *e*_*α*_ is small (e.g., smaller than 30°), a small-angle turning maneuver (e.g., MPF turning) will be triggered; and (5) when the goal is out of vision, the robotic fish will switch to search the goal through 360° turn.

It is worthwhile to remark that, in essence, the vision-based navigation consists of finding the function that maps from specific visual information to a CPG control parameter set $\left\{\omega_{i},\Gamma_{i},{\overset{-}{\theta }}_{i}\right\}$. A direct coupling of visual perception to locomotion requires a huge knowledge base, which is computationally intense and too slow for real-time control applications. Integration of embedded vision, CPG-based control, and fuzzy logic may be a feasible solution to these goal-oriented tasks. Within this framework, the robotic fish will be able to execute goal-oriented aquatic missions.

## 4. Experiments and Results

In order to evaluate the proposed mechatronic design and swimming performance of the embedded vision guided robotic fish with multiple control surfaces, extensive experiments were conducted in an indoor swim tank with clear water. During experiments, unless otherwise stated, the data points and error bars shown in subsequent figures were the averages and standard deviations of three runs. Currently, the robotic fish has successfully achieved autonomous obstacle avoidance and 3D swimming. Available propulsive modes include the BCF-type swimming, the MPF-type swimming, and their combination. More quantitative results will be provided below.

### 4.1. Experimental Results

**Figure 9:**
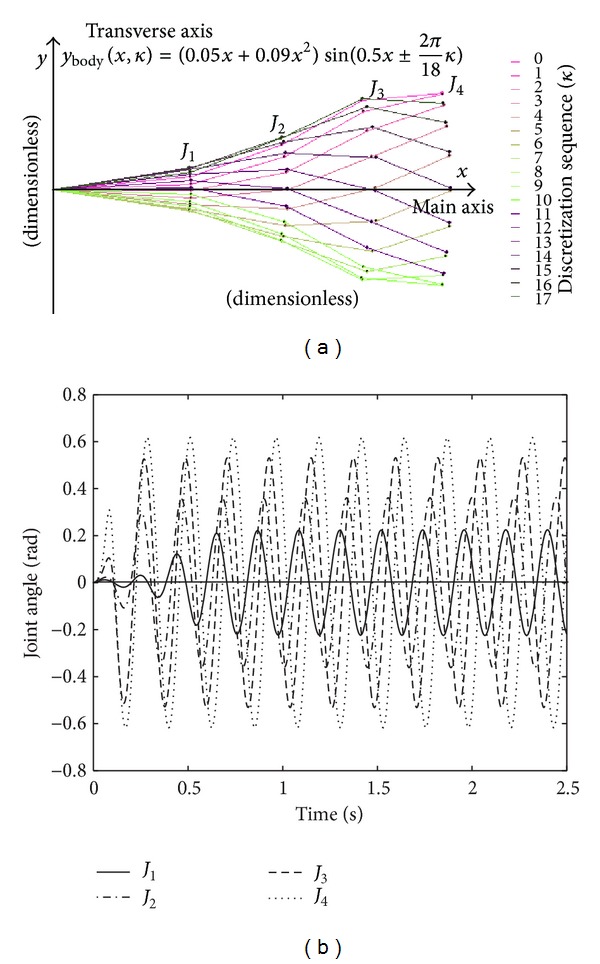
Comparative oscillatory signals for the BCF-type swimming. (a) Oscillatory sequence of *J*_1_–*J*_4_ in the fish body wave fitting. (b) Oscillatory angles of *J*_1_–*J*_4_ in the CPG controller.

**Figure 10:**
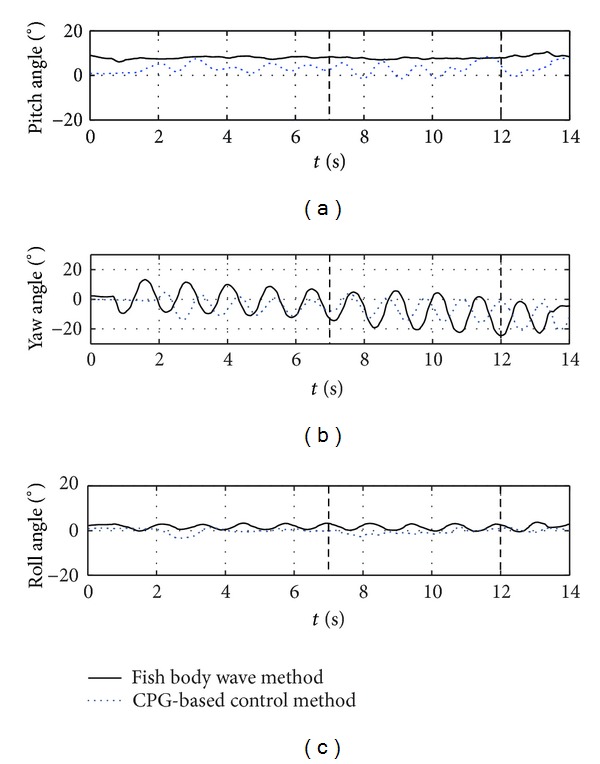
Stability comparison of the CPG-based control method against the fish body wave method in the BCF-type swimming.

The first experiment concerned the stability comparison of the CPG-based control method against the sine-based fish body wave method in the BCF-type swimming.  Figure 9 shows the comparative oscillatory signals of *J*_1_–*J*_4_ calculated from (2) and (4), respectively. The amplitudes of two signals increase monotonically from head to tail. It is worth noting that the caudal fin functions as a principal propulsor in carangiform swimming. Since the caudal fin is rigidly attached to *J*_1_ via the peduncle, the driving signal of *J*_1_ should be carefully devised for highlighting the propulsive effect of the caudal fin. To this end, a minor phase lag is maintained between *J*_1_ and *J*_2_ (see Figure 9(b)). With the oscillatory signals as the joint inputs of the robotic fish, Figure 10 plots the measured pitch, yaw, and roll angles in the world reference frame. During testing, the oscillatory amplitude suddenly increased at 7 s, whereas the amplitude climbed steadily after 12 s. In contrast with the fish body wave method, the CPG-based control method obtained increased pitch and roll stability, yet without loss of yaw stability. Thanks to the intrinsic limit cycle characteristic of the CPGs that is insusceptible to small perturbations, the CPG-based control method provides the possibility to abruptly vary control parameters, while ensuring smooth variations of swimming gaits. Just benefited by this enhanced swimming stability derived from the CPGs, image dithering accompanied by the low-amplitude oscillation of the fish head could be effectively weakened so that steady image capture and visual tracking were available. Note that the CPG-based control method was applied in the following tests.

**Figure 11:**
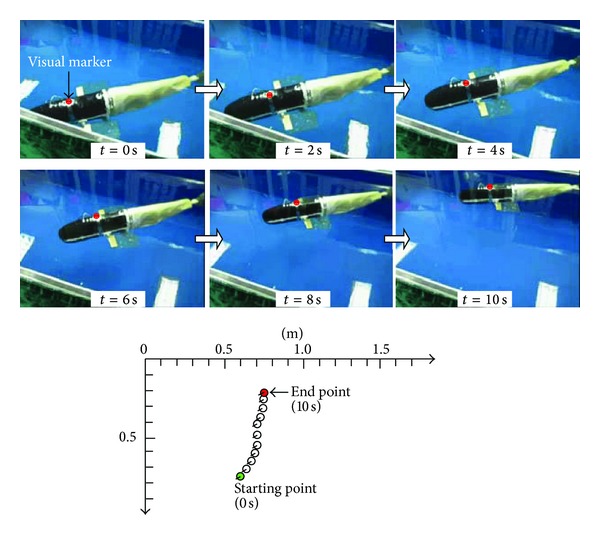
Snapshot sequence and moving trajectory of a sideward swimming gait.

The next experiment was on the newly developed sideward swimming gait. As a UF propulsive mode, the pelvic fin oscillates reciprocally, while the overall fish body keeps straight. A successful example of sideward swimming is illustrated in Figure 11. The average speed attained over an extended period of 10 s was 6.5 ± 0.4 cm/s (mean ± SD) when *ω*_7_ = 20 rad/s. Since the surface of the used pelvic fin (hydrofoil) was relatively small, unlike the large caudal fin, the produced side forces hardly thrust the fish body at a comparable speed. However, this sideward swimming can be blended into normal swimming gaits, allowing for obstacle negotiation or more delicate maneuvers in confined and cluttered spaces.

**Figure 12:**
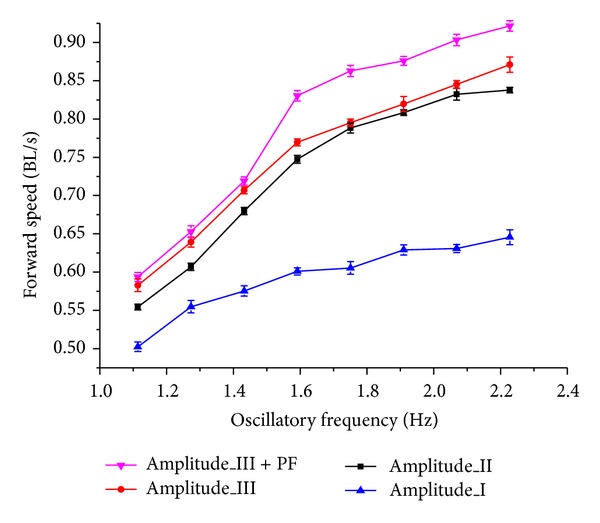
Speed test of the BCF swimming by changing the oscillatory frequency and amplitude.

A third experiment was performed to evaluate the propulsive speeds among different swimming modes. Fish modulate their speeds depending on a hybrid of oscillatory frequency and amplitude of the involved control surfaces. Similar methods can be adopted in the speed control of the robotic fish performing the BCF, the PF, or the BCF + PF swimming. As a demonstration, four swimming cases with various amplitudes were examined. Notice that length-specific speeds expressed as body lengths per second (BL/s) are applied in order to be comparable with other fish robots. The following relationship was also maintained during testing: Amplitude_III = *k*_*a*_ Amplitude_II = *k*_*a*_^2^ Amplitude_I, where *k*_*a*_ = 1.33.  Figure 12 shows the obtained results in the BCF-type swimming: the forward speed directly increased with the oscillatory amplitude and frequency till the actuators peaked their speed limits. Compared with the BCF swimming termed “Amplitude I”, speeds were increased by 22.83 ± 9.10% and 26.58 ± 7.73% in “Amplitude II” and “Amplitude III”, respectively. Remarkably, a combination of the BCF-type and the PF-type termed “Amplitude III+PF” in Figure 12 attained an enhanced speed over the entire range of oscillatory frequency. It is estimated that a speed increase of 33.34 ± 11.18% was obtained in the “Amplitude III+PF” by contrast with the “Amplitude I”, while a speed profit of 5.22 ± 2.85% was even earned relative to the “Amplitude III”. The induced speed increase in the “Amplitude III + PF” implies the cooperative capability of control surfaces in achieving a higher speed. The maximum swimming speed of 0.71 m/s (equivalent to 1.04 BL/s) was obtained at a higher frequency of 3.5 Hz.

**Figure 13:**
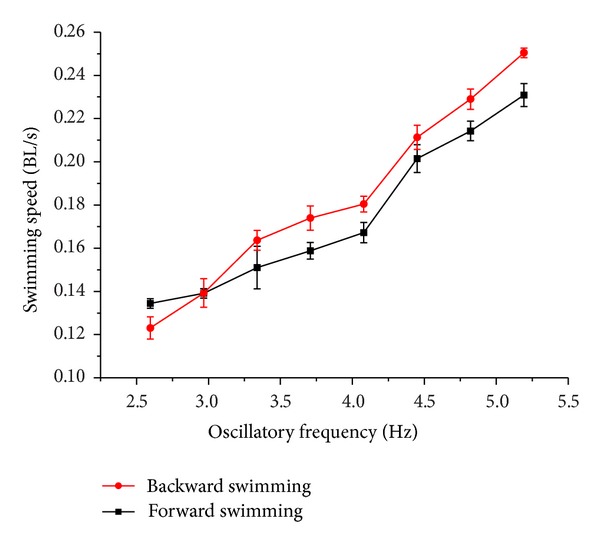
Speed test of the PF swimming by changing the oscillatory frequency.

At the same time, the robotic fish could swim forward or backward by flapping the pectoral fins reciprocally and fleetingly, that is, perform labriform swimming. As shown in Figure 13, the speed of the PF-type swimming also rose over the plotted frequency region but with a relatively low value compared to the BCF-type swimming. This suggests the body shape of the developed robotic fish is more appropriate to carangiform swimming rather than labriform swimming as far as speed is concerned. Notice also that some speed difference (positive or negative) could be detected in the same oscillatory frequency for the PF-type forward and backward swimming, but the difference was not so significant. Despite the same oscillatory frequencies and amplitudes being adopted, entirely consistent results were hard to obtain primarily due to differences of anterior-posterior body shape.

The fourth experiment concerned the exploration of the turning maneuvers. The dependence of the obtained turning radius and angular speed on the angular bias ${\bar{\theta }}_{1}={\bar{\theta }}_{2}$ is summarized in Table 3, where the angular bias was superposed on the rhythmically oscillated body joints (*J*_1_ and *J*_2_) to induce turns. According to the experimental data, increasing the angular bias will shorten the turning radius (represented by body length independent of body size, BL for short) and achieve a larger angular speed (represented by rad/s) simultaneously. Statistical analysis showed that the turning radius was decreased by 17.13 ± 4.95%, whereas the angular speed was increased by 19.33 ± 12.58% in the coordinated BCF + PF turns (where the left and right pectoral fins oscillate out of phase), compared with the BCF turns. Overall, the coordinated BCF + PF turning mode performed better than the BCF turning mode.

In another braking testing with an initial speed of around 0.4 m/s (equivalent to 0.59 BL), the robotic fish stopped in 2.3 s with a cooperative operation of the pectoral fins, the dorsal fin, and the pelvic fin (typically holding the fins vertical to the main axis plane), whereas a longer 5.2 s was needed to halt the fish without any braking measure. This experiment partly highlighted the need for coordinated control surfaces in rapid brake.

**Figure 14:**
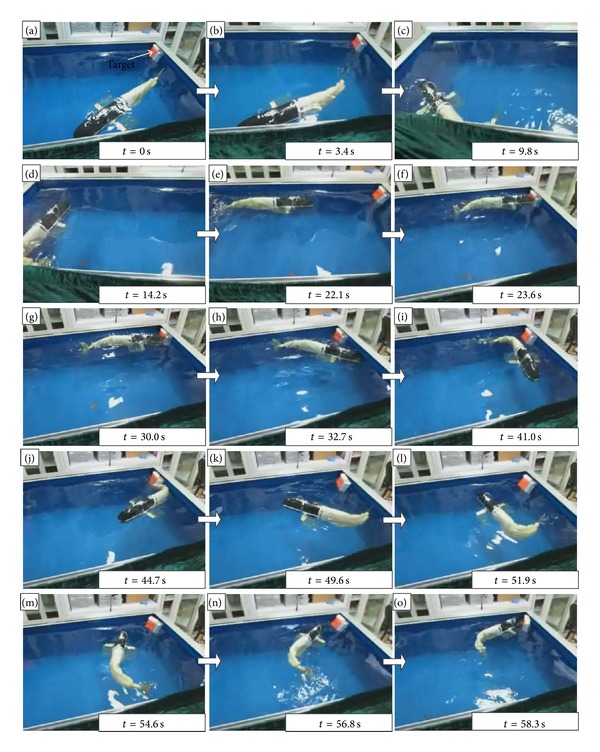
Snapshot sequence of an overall target search and tracking.

**Figure 15:**
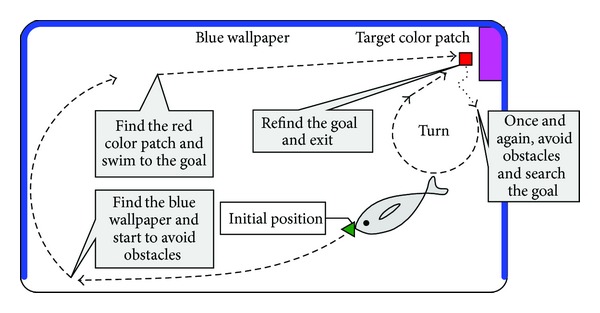
Schematic drawing of the moving trajectory correlating to Figure 14.

At last, the vision-based target search and tracking was examined. In this experiment, the target defined as a red color patch was initially out of the sight of the robotic fish, for example, behind the fish head. Three sides of the swim tank covered with blue wallpaper were considered as the obstacles. The robotic fish was required to find and further reach the target, while swimming and negotiating obstacles, only with the aid of a monocular embedded vision. Notice that all algorithms are embedded into the physical robot and tested without human interruption.  Figure 14 illustrates a complete snapshot sequence of target search and tracking, whose trajectory is schematically plotted in Figure 15. The robotic fish firstly cruised autonomously until it detected the obstacle. Then, the fish turned right, followed by a 180° turn. Afterwards, the target patch came into view of the fish. Thus, the fish swam to the goal directly till it touched the target. This process lasted about 23.6 s, corresponding to Figures 14(a)–14(f). After several drifts of the fish body, the target was again out of the sight of the fish at about 30 s. Since the target was in the corner, the fish had to turn away. With a 360° turn, the fish refound and reached the goal at approximately 58.3 s (see Figures 14(g)–14(o)). Afterwards, the search and tracking task ended and switched to other tasks. The movements of the robotic fish were relatively steady and smooth throughout the testing process, which partially validates the feasibility of the formed navigation strategy. We remark that the test results are acceptable for a goal-directed task. If a binocular stereo vision is imported and an area of open water is available, the embedded vision guided robotic fish will perform better and expand applicability to underwater exploration and other relatively complex real-world aquatic tasks.

### 4.2. Discussion

Fish are masters of multidisciplined scientific synergy. As a demonstration of coordinated control surfaces, the developed robotic prototype primarily validates the mechanics and control mechanism. Specifically, the propulsive speed of the BCF + PF swimming mode was increased by 33.34 ± 11.18% compared with the small-amplitude BCF swimming and by 5.22 ± 2.85% compared with the large-amplitude BCF swimming; the turning radius was decreased by 17.13 ± 4.95%, whereas the angular speed was increased by 19.33 ± 12.58% in the BCF + PF turns. In contrast with our previous work on the CPG-governed dolphin robot with a similar mechanical structure [22], the onboard sensory feedback is incorporated into the CPG-based control loop to enable practical autonomous navigation. The pelvic fin is first imported to add a dynamic quality to sideward movements. Compared to the CPG-based control of a four-joint robotic fish shown in [28], a test result on the stability comparison of the CPG-based control method against the sine-based fish body wave method is given, supporting superior stability against small perturbations in the CPG-based swimming control. Moreover, as opposed to the exploration task oriented thunniform robotic fish shown in [5], whose maximum swimming speed is 1.36 m/s (approximately 1.1 BL/s) and turning radius is 1.75 m (approximately 1.42 BL), the developed multifin robotic fish achieved a comparable speed (up to 1.04 BL/s) yet a turning radius as small as 0.18 BL. This significantly improved turning performance that further verifies the necessity of the coordinated use of multiple control surfaces. However, we should admit that the obtained performance of the robotic fish is fairly inferior to its biological counterpart. There is still vast room for performance improvement and control implementation at a real-time level.

Another issue to be addressed is underwater vision. For robotic fish, vision may be treated as sensory feedback and as a direct information source identifying the environments. With embedded vision integrated in the CPG-based feedback control, the robotic fish can adapt its swimming gait. The coupling of visual perception to the CPG-based control is crucial for autonomous and adaptive control and has received more and more attention in the recent years [14]. However, how to effectively link specific visual information with a CPG control parameter set is a topic worthy of further investigation, since CPG parameter tuning is difficult in itself. In addition, more realistic aquatic visual scenarios (e.g., turbid and wavy waters in outdoor environments, like river and sea) should be considered in order to expedite practical applications of the robotic fish as a mobile underwater platform.

## 5. Conclusions and Future Work

In this paper, we have presented the overall design and control of a novel robotic fish with multiple control surfaces and an embedded vision system. Considering the mechatronic characteristics of the robotic fish and the aquatic environment, a bioinspired CPG-based control enabling online swimming gait generation is adopted to govern multiple control surfaces within a unified self-propelled platform. At the same time, a set of monocular vision algorithms is employed to explore underwater environments in real time. The coupling of onboard visual perception to the CPG-based control enables the robot to perform goal-directed swimming. The results obtained have confirmed that the proposed mechatronic design and control methods are effective. In particular, the experimental verification of a slightly enhanced cruising speed and a fairly improved turning maneuverability due to multiple hydropropulsion surfaces, as well as the combination of BCF and PF swimming is new. The successful implementation of the goal-directed search and tracking reveals promising prospects of the robotic fish serving as a mobile sensing and exploration device.

The ongoing and future work will concentrate on continuing to synthetically improve the mechatronic system and to intensively explore coordinated swimming control via multiple control surfaces coupled with high-speed embedded vision in outdoor environments like river and sea.

## Figures and Tables

**Table 1:** The relations between swimming gaits and control surfaces.

Swimming gaits	Control surfaces	Control parameters
BCF forward	Body + caudal fin	*ω*_*i*_, Γ_*i*_ (*i* = 1,2,…, 4)
Hybrid forward	Body + caudal fin + PF	*ω*_*i*_, Γ_*i*_ (*i* = 1,2,…, 6)
MPF forward	PF	*ω*_*i*_, Γ_*i*_ (*i* = 5,6)
MPF backward	PF	*ω*_*i*_, Γ_*i*_, ${\overset{-}{\theta }}_{i}$ (*i* = 5,6)
BCF turning	Body + caudal fin	*ω*_*i*_, Γ_*i*_, ${\overset{-}{\theta }}_{i}$ (*i* = 1,2,…, 4)
MPF turning	PF and/or pelvic fin	*ω*_*i*_, Γ_*i*_, ${\overset{-}{\theta }}_{i}$ (*i* = 5,6, 7)
Hybrid turning	All involved	*ω*_*i*_, Γ_*i*_, ${\overset{-}{\theta }}_{i}$ (*i* = 1,2,…, 7)
Sideward swimming	Pelvic fin	*ω*_*i*_, Γ_*i*_, ${\overset{-}{\theta }}_{i}$ (*i* = 7)
Diving/surfacing	PF or BCF + PF	*ω*_*i*_, Γ_*i*_, ${\overset{-}{\theta }}_{i}$ (*i* = 1,2,…, 6)
Braking	All involved	*ω*_*i*_, Γ_*i*_, ${\overset{-}{\theta }}_{i}$ (*i* = 1,2,…, 4)

**Table 2:** CPG parameter values applied to the robotic fish.

Parameters	Value
*ω*	*ω*_*i*_ ∈ [0, 52)
Γ	Γ_i_ ∈ [0,60]
*a*	*a*_1_ = 0.3, *a*_2_ = −0.2
*b*	*b*_1_ = 0.2, *b*_2_ = −0.1
*γ*_*i*_	0.0
*u*_*b*_^*i*^	30

**Table 3:** Turning performance characterized by angular bias in different gaits at 2 Hz.

	Angular bias (°)	15	30	45
BCF	Turning radius (BL)	0.84 ± 0.03	0.62 ± 0.03	0.23 ± 0.04
	Angular speed (rad/s)	0.65 ± 0.04	0.87 ± 0.02	1.58 ± 0.07
BCF + PF	Turning radius (BL)	0.74 ± 0.03	0.51 ± 0.03	0.18 ± 0.02
	Angular speed (rad/s)	0.87 ± 0.04	0.97 ± 0.04	1.78 ± 0.05

## References

[1] Lepora NF, l Verschure P, Prescott TJ (2013). The state of the art in biomimetics. *Bioinspiration & Biomimetics*.

[2] Triantafyllou MS, Triantafyllou GS (1995). An efficient swimming machine. *Scientific American*.

[3] Bandyopadhyay PR, Beal DN, Menozzi A (2008). Biorobotic insights into how animals swim. *Journal of Experimental Biology*.

[4] Yu J, Tan M, Wang S, Chen E (2004). Development of a biomimetic robotic fish and its control algorithm. *IEEE Transactions on Systems, Man, and Cybernetics, Part B*.

[5] Liang J, Wang T, Wen L (2011). Development of a two-joint robotic fish for real-world exploration. *Journal of Field Robotics*.

[6] Liu J, Hu H (2010). Biological inspiration: from carangiform fish to multi-joint robotic fish. *Journal of Bionic Engineering*.

[7] Yu J, Wang M, Tan M, Zhang J (2011). Three-dimensional swimming. *IEEE Robotics and Automation Magazine*.

[8] Sfakiotakis M, Lane DM, Davies JBC (1999). Review of fish swimming modes for aquatic locomotion. *IEEE Journal of Oceanic Engineering*.

[9] Lauder GV, Drucker EG (2004). Morphology and experimental hydrodynamics of fish fin control surfaces. *IEEE Journal of Oceanic Engineering*.

[10] Helfman GF, Collette BB, Facey DE, Bowen BW (2009). *The Diversity of Fishes: Biology, Evolution, and Ecology*.

[11] Lachat D, Crespi A, Ijspeert AJ BoxyBot: a swimming and crawling fish robot controlled by a central pattern generator.

[12] Kodati P, Hinkle J, Winn A, Deng X (2008). Microautonomous robotic ostraciiform (MARCO): hydrodynamics, design, and fabrication. *IEEE Transactions on Robotics*.

[13] Collin SP, Marshall NJ (2003). *Sensory Processing in Aquatic Environments*.

[14] Gay S, Dégallier S, Pattacini U, Ijspeert A, Victor JS Integration of vision and central pattern generator based locomotion for path planning of a non-holonomic crawling humanoid robot.

[15] Zou W, Yu J, Xu D (2009). Development of the embedded vision system: a survey. *Measurement and Control*.

[16] Wang W, Yu J, Wang M, Ding R Mechanical design and preliminary realization of robotic fish with multiple control surfaces.

[17] Yu J, Wang M, Wang W, Tan M, Zhang J Design and control of a fish-inspired multimodal swimming robot.

[18] Hove JR, O’Bryan LM, Gordon MS, Webb PW, Weihs D (2001). Boxfishes (Teleostei: Ostraciidae) as a model system for fishes swimming with many fins: kinematics. *Journal of Experimental Biology*.

[19] Mackean DG Fish—structure and function. http://www.biology-resources.com/fish-01.html.

[20] Salumäe T, Kruusmaa M (2011). A flexible fin with bio-inspired stiffness profile and geometry. *Journal of Bionic Engineering*.

[21] Ijspeert AJ (2008). Central pattern generators for locomotion control in animals and robots: a review. *Neural Networks*.

[22] Yu J, Wang M, Tan M, Zhang J (2011). Three-dimensional swimming. *IEEE Robotics and Automation Magazine*.

[23] Wang M, Yu J, Tan M Parameter design for a central pattern generator based locomotion controller.

[24] Wu Z, Yu J, Tan M CPG parameter search for a biomimetic robotic fish based on particle swarm optimization.

[25] Kocak DM, Caimi FM (2005). The current art of underwater imaging—with a glimpse of the past and vision of the future. *Marine Technology Society Journal*.

[26] Chen J, Xiao G, Gao F, Zhou H, Ying X Vision-based perceptive framework for fish motion.

[27] Comaniciu D, Ramesh V, Meer P (2003). Kernel-based object tracking. *IEEE Transactions on Pattern Analysis and Machine Intelligence*.

[28] Zhao W, Hu Y, Zhang L, Wang L (2009). Design and CPG-based control of biomimetic robotic fish. *IET Control Theory and Applications*.

